# VWF/ADAMTS13 ratio as a potential biomarker for early detection of hepatocellular carcinoma

**DOI:** 10.1186/s12876-019-1082-1

**Published:** 2019-10-21

**Authors:** Hiroaki Takaya, Tadashi Namisaki, Mitsuteru Kitade, Kosuke Kaji, Keisuke Nakanishi, Yuki Tsuji, Naotaka Shimozato, Kei Moriya, Kenichiro Seki, Yasuhiko Sawada, Soichiro Saikawa, Shinya Sato, Hideto Kawaratani, Takemi Akahane, Ryuichi Noguchi, Masanori Matsumoto, Hitoshi Yoshiji

**Affiliations:** 1 Third Department of Internal Medicine, Nara Medical University, Shijo-cho 840, Kashihara, Nara, 634-8522 Japan; 2 Department of Blood Transfusion Medicine, Nara Medical University, Kashihara, Nara, 634-8522 Japan

**Keywords:** ADAMTS13, VWF, Biomarker, HCC, Early diagnosis

## Abstract

**Background:**

To investigate the von Willebrand factor to ADAMTS13 ratio as a potential biomarker for early detection of hepatocellular carcinoma (HCC) in cirrhosis.

**Methods:**

Serum levels of alpha-fetoprotein, des-γ-carboxy prothrombin, *Lens culinaris* agglutinin-reactive fraction of alpha-fetoprotein (alpha-fetoprotein-L3%), vascular endothelial growth factor, and vascular endothelial growth factor receptor-2, as well as the plasma levels of von Willebrand factor antigen (von Willebrand factor: Ag) and ADAMTS13 activity (ADAMTS13:AC), were evaluated in 41 cirrhotic patients with HCC undergoing radiofrequency ablation and in 20 cirrhotic patients without HCC. The diagnostic accuracy of each biomarker was evaluated using the receiver operating characteristic curve analysis.

**Results:**

The von Willebrand factor: Ag and von Willebrand factor: Ag/ADAMTS13:AC ratios were significantly higher in cirrhotic patients with HCC than in those without HCC (*p* < 0.05 and *p* < 0.01, respectively), whereas ADAMTS13:AC was significantly lower in those with HCC than those without HCC (p < 0.05). However, no relationship was observed between the von Willebrand factor: Ag/ADAMTS13:AC ratio and serum tumor markers such as alpha-fetoprotein, des-γ-carboxy prothrombin, and alpha-fetoprotein-L3%. Multivariate regression analysis identified von Willebrand factor: Ag/ADAMTS13:AC ratio and alpha-fetoprotein-L3% as significant factors of HCC development. Receiver operating characteristic analysis showed that the von Willebrand factor: Ag/ADAMTS13:AC ratio and alpha-fetoprotein-L3% had a better performance than alpha-fetoprotein, des-γ-carboxy prothrombin, alpha-fetoprotein-L3%, vascular endothelial growth factor, and vascular endothelial growth factor receptor-2, von Willebrand factor: Ag, and ADAMTS13:AC. The von Willebrand factor: Ag/ADAMTS13:AC ratio was exclusively correlated with tumor volume and stage as well as serum vascular endothelial growth factor levels.

**Conclusions:**

The von Willebrand factor: Ag/ADAMTS13:AC ratio can potentially serve as a novel biomarker for early diagnosis of HCC in cirrhotic patients.

## Background

Hepatocellular carcinoma (HCC) represents the sixth most common cancer worldwide [1] and is the second leading cause of cancer-related death [2, 3]. Japan has one of the highest incidences of HCC in the world [1, 4]. Therefore, the diagnosis of very early stage HCC (defined as a single nodule sized ≤2 cm) is a vital prerequisite for optimizing the treatment outcomes; however, until date, it remains challenging. The professional society guidelines from the European Association for the Study of the Liver [5] and American Association for the Study of Liver Diseases [6] encourage surveillance using ultrasonography at every 6 months in patients with cirrhosis. However, they do not agree with the addition of alpha fetoprotein (AFP) as an adjunct surveillance test. The Japan Society of Hepatology Consensus-Based Clinical Practice Guidelines for the Management of HCC recommends the use of three tumor markers AFP, des-γ-carboxy prothrombin (DCP), and alpha-fetoprotein-L3% (AFP-L3) in combination with ultrasonography [7]. This approach has contributed to increase the detection of small HCCs in Japan in comparison to that in Europe and America. Furthermore, AFP-L3% has been identified as a biomarker for the early detection of HCC due to its higher specificity in comparison with that of AFP [8]. The combination use of AFP-L3 and DCP plus ultrasonography achieved a sensitivity of approximately 85% and a specificity of approximately 95% [9], whereas AFP yielded no additional benefit to ultrasonography [10]. However, AFP-L3 is not routinely used outside of Japan. Therefore, universally available biological markers for the early diagnosis of HCC are urgently needed to improve the survival rate among patients with cirrhosis.

The tumor microenvironment, notably including the surrounding blood vessels; the hepatic non-parenchymal cells, such as Kupffer cells, hepatic stellate cells (HSCs), liver sinusoidal endothelial cells; and the diverse types of lymphocytes, plays a crucial role in tumor initiation and progression in HCC [11]. A disintegrin-like and metalloproteinase with thrombospondin type-1 motifs 13 (ADAMTS13) is predominantly produced in HSCs and cleaves newly secreted, unusually large von Willebrand factor (VWF) multimers (UL-VWFM) on the endothelial surface under high shear stress [12–14]. An imbalance between ADAMTS13 activity and UL-VWFM, formed by endothelial cell (EC) secretion of VWF from the endothelial surface, triggers thrombosis by inducing platelet adhesion and aggregation [15]. This marked imbalance between VWF antigen (VWF:Ag) and ADAMTS13 activity (ADAMTS13:AC) is related closely to impaired hepatic functional reserve in cirrhotic patients [16, 17]. The components of the coagulation cascade regulate various steps in tumor initiation, progression, and metastasis [18, 19]; therefore, coagulation-related factors could be molecular targets for the diagnosis of patients with HCC. The aim of this study was to investigate the VWF and ADAMTS13 as potential biomarkers for the early detection of HCC in cirrhotic patients.

## Methods

### Patients

A retrospective review of medical records was performed for 61 consecutive patients aged ≥20 years with cirrhosis, of whom 41 (67.2%) developed HCC and visited the Nara Medical University, Kashihara, Nara, Japan between April and November 2016. Patients with initial hypervascular HCCs who were diagnosed using dynamic contrast-enhanced CT (DCE-CT), DCE-MRI, or DCE-ultrasound (DCE-US) were enrolled in the present study. Percutaneous radio frequency ablation (RFA) was performed for all patients according to the Japan Society of Hepatology Consensus-Based Clinical Practice Guidelines for HCC management [20]. All patients underwent blood examination for AFP, DCP, and AFP-L3% before the RFA procedure. Patients with infection, thrombosis, ascites, hepatic encephalopathy, or uncontrolled gastroesophageal varices were excluded. Patients who received anticoagulants were also excluded. Written consent was obtained from all patients who agreed to participate in the study when they were admitted to hospital and treated with RFA. The local ethics committee of Nara Medical University approved this study, and the study was performed in accordance with the ethical standards established in the Declaration of Helsinki. Informed consent was obtained from all the participants.

### Measurement of the three tumor markers

Blood samples were obtained from cirrhotic patients with HCC prior to RFA. The serum AFP level was determined by enzyme-linked immunosorbent assay using a commercially available kit (ELISA-AFP; International Reagents, Kobe, Japan) [21, 22]. The serum DCP level was determined by sensitive enzyme immunoassay (Eitest PIVKA-II kit; Eisai Laboratory, Tokyo, Japan), according to the manufacturer’s instructions [23, 24]. The serum AFP-L3 levels were measured using lectin affinity electrophoresis coupled with antibody affinity blotting (AFP Differentiation Kit L; Wako Pure Chemical Industries, Ltd., Osaka, Japan) and were finally expressed as a percent of AFP-L3 (AFP-L3 level/total AFP level × 100) [25–27] .

### Measurement of the serum vascular endothelial growth factor (VEGF) and VEGF receptor-2 (VEGFR2) levels

Serum VEGF and VEGFR2 levels were determined using commercially available ELISA kits (Quantikine Human VEGF Immunoassay and Quantikine Human VEGF R2 Immunoassay; R&D Systems, Minneapolis, MN, USA) according to the manufacturer’s instructions [28, 29].

### Determination of plasma levels of VWF antigen and ADAMTS13 activity

Blood samples were obtained from patients at the time of admission, during their hospital stay, or during regular outpatient treatment and were stored in plastic tubes containing 0.38% volume of sodium citrate. Platelet-poor plasma was prepared by centrifuging at 3000×g at 4 °C for 15 min and was stored in aliquots at − 80 °C until analysis. The sensitive chromogenic ELISA (Kainos Laboratories Inc., Tokyo, Japan) was used to determine Plasma ADAMTS13:AC [30]. The normal value for ADAMTS13:AC was 99 ± 22%. Plasma VWF:Ag was measured by sandwich ELISA using a rabbit anti-human VWF polyclonal antiserum (Dako, Glostrup, Denmark). The normal value for VWF:Ag is 102% ± 33% [31].

### Statistical analysis

The Mann–Whitney U test was used to analyze the differences between cirrhotic patients with and without HCC. The Spearman’s rank test was used to calculate correlations. Fisher’s exact test was used to analyze categorical data. The data were expressed as mean ± standard deviation. Univariate analysis and multivariate logistic regression with stepwise variable selection were used to determine the factors associated with early detection of HCC. The diagnostic accuracy of biomarkers for the early diagnosis of HCC [sensitivity, specificity and area under the curve (AUC)] was determined using the area under the receiver operating characteristic (ROC) curve [32, 33]. A two-tailed *p* value of less than 0.05 was considered significant. All analyses were carried out using EZR (Saitama Medical Center, Jichi Medical University), a graphical user interface for R (The R Foundation for Statistical Computing, version 2.13.0). Specifically, EZR is a modified version of R commander (version 1.6–3) that includes statistical functions that are frequently used in biostatistics [34].

## Results

### Clinical characteristics of the patients

Table 1 summarizes the clinical characteristics of the 61 cirrhotic patients with and without HCC. The study population had a median age of 74 years and comprised 42 men and 19 women. The causes of liver disease were hepatitis C virus (HCV) (*n* = 28), hepatitis B virus (HBV) (*n* = 16), alcohol abuse (*n* = 8), non-alcoholic steatohepatitis (*n* = 6), and autoimmune hepatitis (*n* = 3). The median age of patients who developed HCC was 78 years. *The* median tumor *size and volume* were 1.6 cm and 4.0 cm^3^, respectively. The numbers of stage 1, 2, and 3 cases were 15, 19, and 7, respectively. The median serum aspartate aminotransferase and alanine aminotransferase levels were 30 IU/L and 27 IU/L, respectively. There were no significant differences between cirrhotic patients with and without HCC in all the characteristics, except for sex and the serum albumin level.

**Table 1:** Baseline characteristics of cirrhotic patients with and without HCC

Variable	Total (*n* = 61)	Patients with HCC (*n* = 41)	Patients without HCC (*n* = 20)	*P* value
Age (years)	74 (67–79)	78 (67–79)	72 (64–76)	0.19
Sex (male/female)	42/19	32/9	10/10	0.04
HCV/HBV/Alcohol/NASH/AIH	28/16/8/6/3	17/11/6/6/1	11/5/2/0/2	0.26
Albumin (g/dL)	4.1 (3.6–4.4)	3.9 (3.4–4.1)	4.4 (4.2–4.6)	0.000004
Total bilirubin (mg/dL)	0.9 (0.7–1.2)	0.9 (0.7–1.2)	0.85 (0.8–1.2)	0.88
Aspartate aminotransferase (IU/L)	30 (22–41)	30 (21–47)	29.5 (26–34)	0.68
Alanine aminotransferase (IU/L)	27 (19–47)	33 (21–47)	22 (16–36)	0.12
alkaline phosphatase (IU/L)	315 (233–432)	315 (237–427)	314 (230–483)	0.93
γ-glutamyl transpeptidase (IU/L)	33 (24–56)	32 (24–82)	34 (23–45)	0.58
Prothrombin time (%)	78 (69–87)	75 (70–84)	81 (75–85)	0.21
Child-Pugh score	5.0 (5–6)	5.0 (5–6)	5.0 (5–5)	0.058
Platelet count (×10^4^/μL)	11.9 (9.0–14.8)	11.9 (9.1–15.4)	11.2 (9.0–13.0)	0.52
Tumor size (cm)		1.6 (1.2–2.2)		
Tumor volume (cm^3^)		4.0 (1.7–6.1)		
UICC TNM stage (stage 1/stage 2/stage 3)		15/19/7		
GALAD model (Score, Probability)	1.8 (0.7–3.2), 85 (66–96)	2.4 (1.5–3.4), 92 (81–98)	0.7 (−1.1–1.1), 67 (27–75)	0.00056, 0.00068

Data are expressed as median (Inter Quartile Range)

*P*-values represent comparisons between cirrhotic patients with and without HCC

*HCC* hepatocelullar carcinoma, *HCV* hepatitis C virus, *HBV* hepatitis B virus

*NASH* non-alcoholic steatohepatitis, *AIH* autoimmune hepatitis

*TMN* tumor/node/metastasis

### Differences between patients with and without HCC in tumor markers, GALAD score, angiogenic factors and pro-coagulation markers

**Fig. 1:**
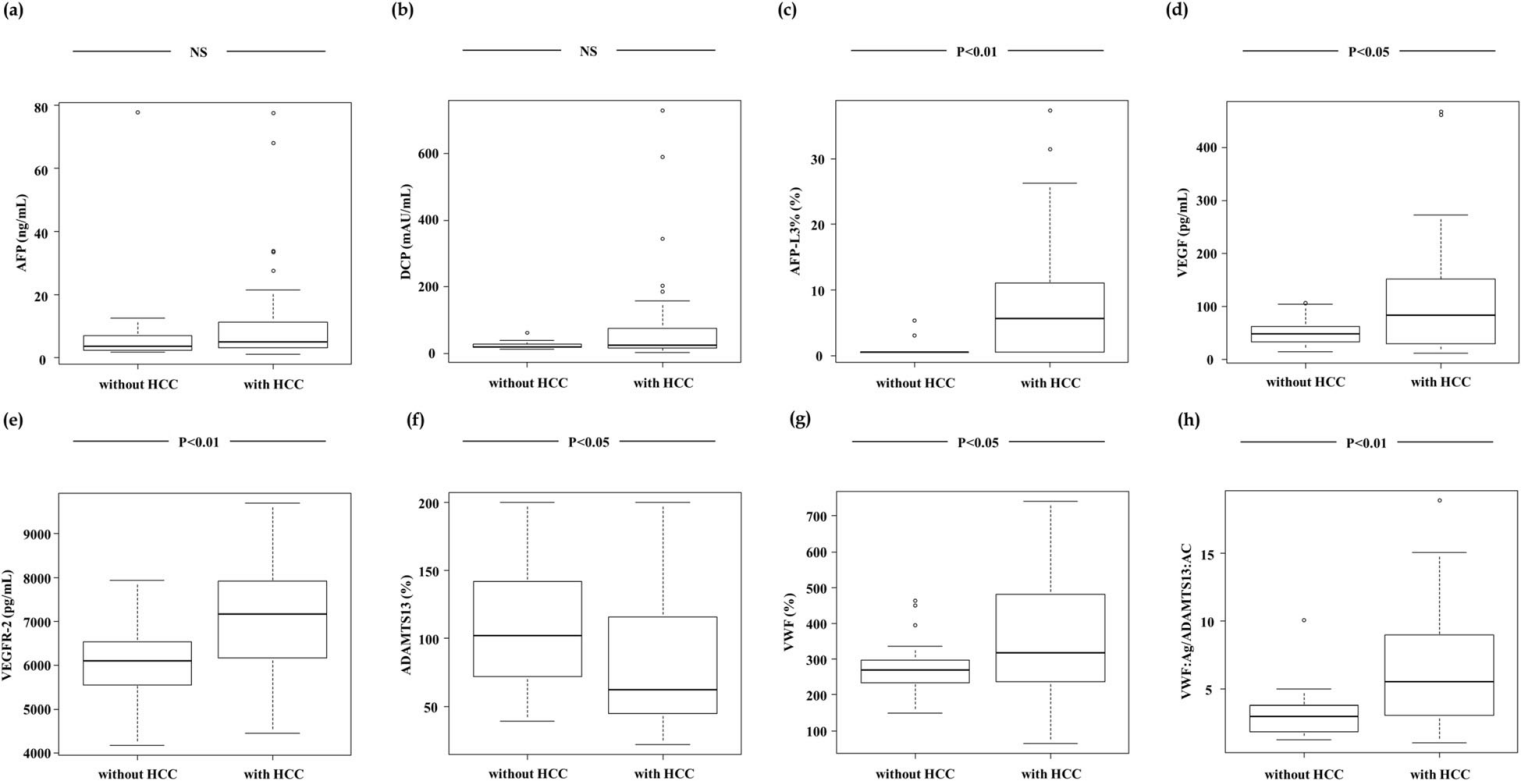
Comparison of serum biomarkers between cirrhotic patients with HCC and those without HCC. The AFP (**a**) and DCP (**b**) levels did not differ significantly between cirrhotic patients with and without HCC. The AFP-L3% (**c**), VEGF (**d**), VEGFR-2 (**e**) levels, as well as the VWF:Ag (**g**) and VWF:Ag/ADAMTS13:AC ratio (**h**), were significantly higher in cirrhotic patients with HCC than in those without HCC. The ADAMTS13:AC (**f**) was significantly lower in patients with HCC than in those without HCC. Asterisks indicate statistically significant differences between the indicated experimental groups (*p* < 0.05, *p* < 0.01). VWF, von Willebrand factor; VWF:Ag, VWF antigen; ADAMTS13, a disintegrin-like and metalloproteinase with thrombospondin type 1 motifs 13; ADAMTS13:AC, ADAMTS13 activity; VEGF, vascular endothelial growth factor; VEGFR-2, VEGF receptor-2; AFP, alpha-fetoprotein; AFP-L3%, *Lens culinaris* agglutinin-reactive AFP; DCP, des-γ-carboxy prothrombin; HCC: hepatocellular carcinoma; NS, not significant

We compared the tumor markers, angiogenic factors, and pro-coagulation markers between cirrhotic patients with and without HCC. The AFP-L3%, GALAD score, VEGF, VEGFR-2 levels, as well as the VWF:Ag and VWF:Ag/ADAMTS13:AC ratio, were significantly higher in cirrhotic patients with HCC than in those without HCC (*p* < 0.01, *p* < 0.01, *p* < 0.05, *p* < 0.01, *p* < 0.05, and *p* < 0.01, respectively) (Fig. 1 c, d, e, g, and h). However, the ADAMTS13:AC was significantly lower in patients with HCC than in those without HCC (*p* < 0.05) (Fig. 1 f). The AFP and DCP levels did not differ significantly between cirrhotic patients with and without HCC (Fig. 1 a and b).

### Correlation of the VWF:ag/ADAMTS13:AC ratio with conventional tumor markers and platelet count

**Fig. 2:**
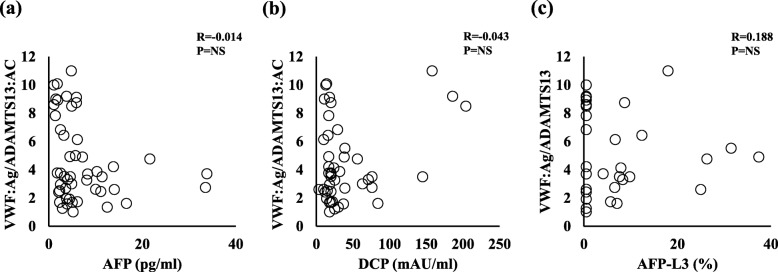
Correlation of the VWF:Ag/ADAMTS13:AC ratio with conventional tumor markers. The VWF:Ag/ADAMTS13:AC ratio is not significantly correlated with the (**a**) AFP level, (**b**) DCP level, and (**c**) AFP-L3%. VWF, von Willebrand factor; VWF:Ag, VWF antigen; ADAMTS13, a disintegrin-like and metalloproteinase with thrombospondin type 1 motifs 13; ADAMTS13:AC, ADAMTS13 activity; AFP, alpha-fetoprotein; AFP-L3%, *Lens culinaris* agglutinin-reactive AFP; DCP, des-γ-carboxy prothrombin; NS, not significant

**Fig. 3:**
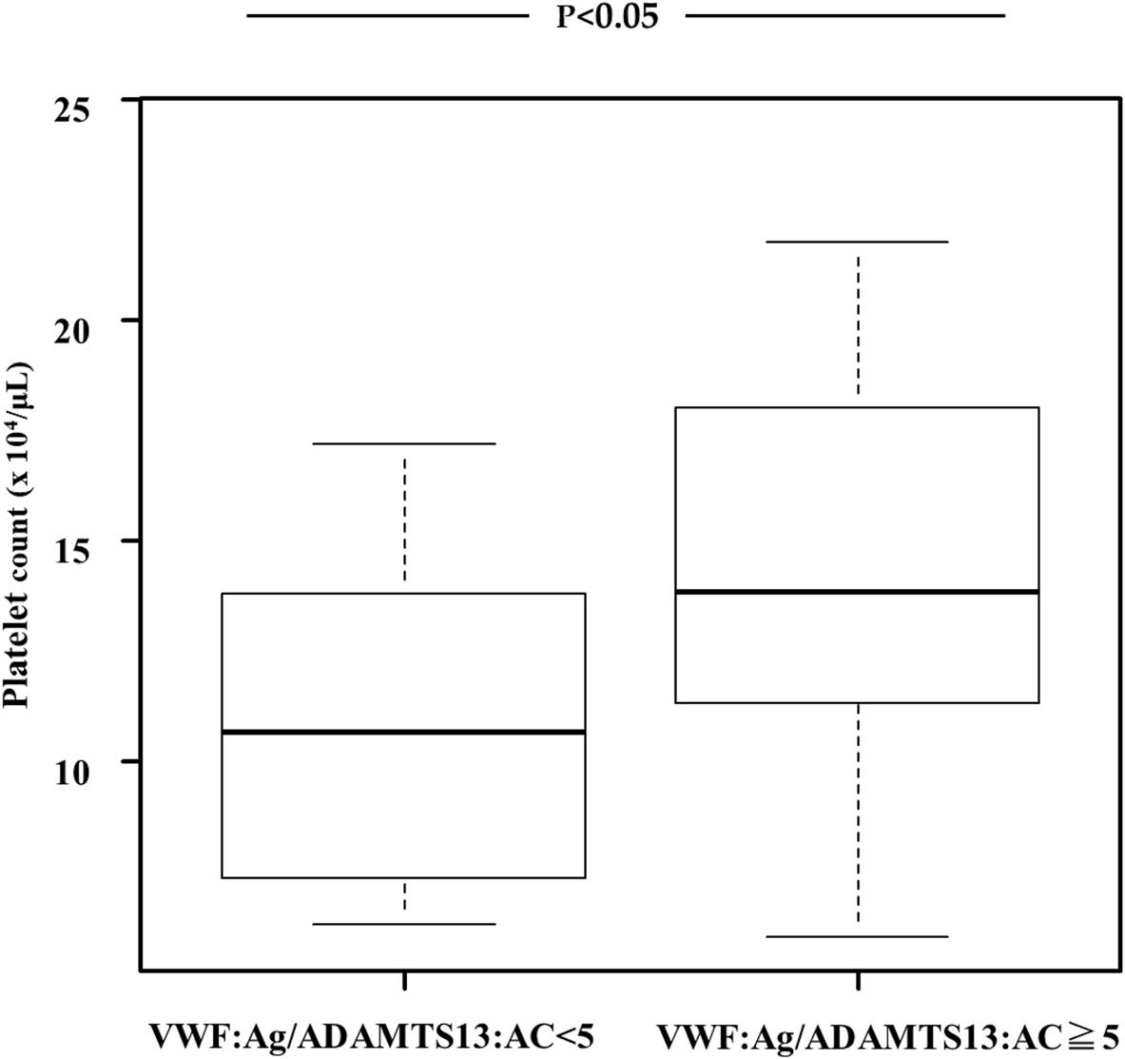
Correlation of the VWF:Ag/ADAMTS13:AC ratio with platelet count. The platelet count is significantly higher in cirrhotic patients with HCC and a VWF:Ag/ADAMTS13:AC ratio ≥ 5 than in those with VWF:Ag/ADAMTS13:AC ratio < 5. Asterisks indicate statistically significant differences between the indicated experimental groups (*p* < 0.05). VWF, von Willebrand factor; VWF:Ag, VWF antigen; ADAMTS13, a disintegrin-like and metalloproteinase with thrombospondin type 1 motifs 13; ADAMTS13:AC, ADAMTS13 activity; HCC, hepatocellular carcinoma

We examined the relationship between the VWF:Ag/ADAMTS13:AC ratio and serum tumor markers, such as AFP, DCP, and AFP-L3%. No relationship was observed between the VWF:Ag/ADAMTS13:AC ratio and any of the three tumor markers (Fig. 2 a, b, and c). The patients were categorized into two, according to the median cutoff VWF:Ag/ADAMTS13:AC ratio (low, < 5 and high, ≥5). The patients with a VWF:Ag/ADAMTS13:AC ratio ≥ 5 had significantly higher platelet count, compared with those with a ratio < 5 (Fig. 3). This indicated that the imbalance between VWF:Ag and ADAMTS13:AC may be linked to platelet hyperaggregability in cirrhotic patients with HCC.

### Diagnostic accuracy of the serum biomarkers for the early diagnosis of HCC

**Fig. 4:**
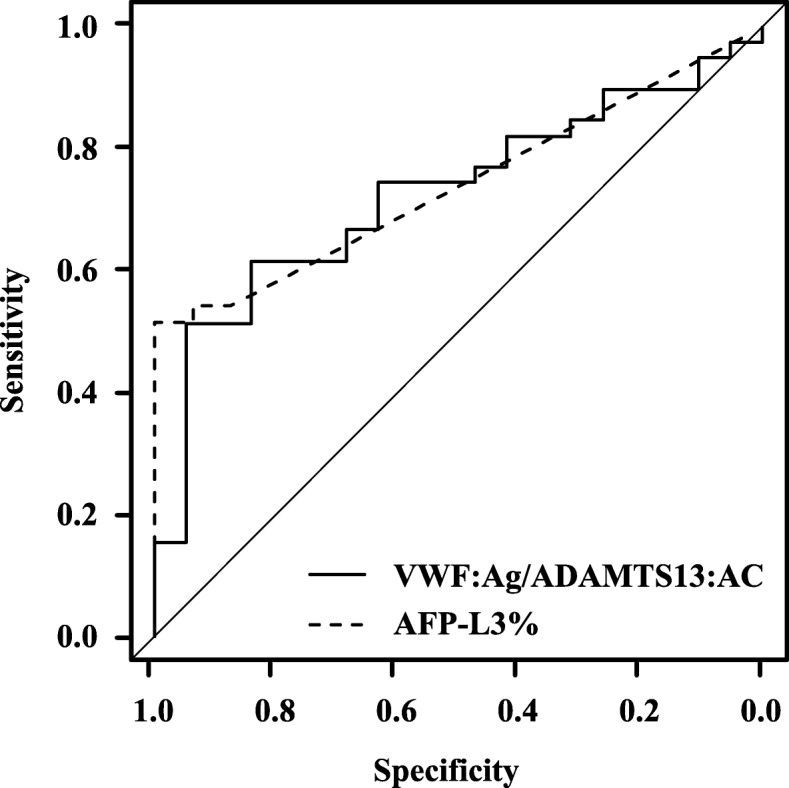
Diagnostic accuracy of serum biomarkers for the early detection of HCC. The diagnostic accuracy of the VWF:Ag/ADAMTS13:AC ratio (Cutoff value 5.5, sensitivity 51%, specificity 95%, and AUC 0.73) is comparable with that of AFP-L3% (Cutoff value 5.7, sensitivity 54%, specificity 93%, and AUC 0.74). VWF, von Willebrand factor; VWF:Ag, VWF antigen; ADAMTS13, a disintegrin-like and metalloproteinase with thrombospondin type 1 motifs 13; ADAMTS13:AC, ADAMTS13 activity; AUC, area under the curve; AFP-L3%, *Lens culinaris* agglutinin-reactive alpha-fetoprotein; HCC, hepatocellular carcinoma

Univariate and multivariate logistic regression analyses confirmed that the VWF:Ag/ADAMTS13:AC ratio and AFP-L3% were significant factors associated with the early detection of HCC in cirrhotic patients (Table 2). ROC analysis showed that the area under the ROC curve of AFP, DCP, AFP-L3%, VEGF, VEGFR2, ADAMTS13:AC, VWF:Ag, and the VWF:Ag/ADAMTS13:AC ratio for the early diagnosis of HCC in patients with cirrhosis was 0.61, 0.58, 0.54, 0.74, 0.67, 0.59, 0.67, 0.63, and 0.73, respectively. The VWF:Ag/ADAMTS13:AC ratio and AFP-L3% showed a better performance than AFP, DCP, VEGF, VEGFR2, ADAMTS13:AC, and VWF:Ag (Table. 3). The diagnostic accuracy of the VWF:Ag/ADAMTS13:AC ratio (Cutoff value 5.5, sensitivity 51%, specificity 95%, and AUC 0.73) is comparable with that of AFP-L3% (Cutoff value 5.7, sensitivity 54%, specificity 93%, and AUC 0.74) (Fig. 4).

**Table 2:** The diagnostic accuracy of biomarkers for early detection of HCC

Variable	OR (95% CI)	*P* value
Unvariable analysis		
AFP > 10 ng/mL	1.01 (0.98–1.05)	0.49
DCP > 40mAU/mL	5.48 (1.10–27.3)	0.038
AFP-L3% > 5%	15.9 (1.89–134.0)	0.011
VEGF > 100 pg/mL	7.06 (1.39–35.9)	0.019
VEGFR2 > 6500 pg/mL	2.46 (0.796–7.63)	0.12
ADAMTS13:AC > 60%	0.31 (0.0874–1.10)	0.082
VWF:Ag > 300%	4.31 (1.30–14.3)	0.017
VWF:Ag/ADAMTS13:AC > 5	8.95 (1.82–44.0)	0.007
Multivariable analysis		
AFP-L3% > 5%	19.5 (1.78–214.0)	0.015
VWF:Ag/ADAMTS13:AC > 5	18.5 (1.64–209.0)	0.018

*HCC* hepatocellular carcinoma, *AFP* alpha fetoprotein, *DCP* des-γ-carboxy prothrombin

AFP-L3%, *Lens culinaris* agglutinin-reactive alpha-fetoprotein

VEGF, vascular endothelial growth factor; VEGFR-2, VEGF receptor-2

ADAMTS13, a disintegrin-like and metalloproteinase with thrombospondin type 1 motifs 13

VWF, von Willebrand factor; ADAMTS13:AC, ADAMTS13 activity

VWF:Ag, von Willebrand factor antigen; VWF:Ag/ADAMTS13:AC, ratio of VWF:Ag to ADAMTS13:AC. CI, confidence interval; OR, odds ratio

**Table 3:** The receiver operating characteristic (ROC) curve of the biomarkers

Variable	Sensitivity	Specificity	AUC
AFP	0.68	0.55	0.61
DCP	0.46	0.85	0.58
AFP-L3%	0.54	0.93	0.74
VEGF	0.47	1.00	0.67
VEGFR2	0.59	0.58	0.59
ADAMTS13:AC	0.56	0.79	0.67
VWF:Ag	0.59	0.75	0.63
VWF:Ag/ADAMTS13:AC	0.51	0.95	0.73

*AFP* alpha fetoprotein, *DCP* des-γ-carboxy prothrombin

AFP-L3%, *Lens culinaris* agglutinin-reactive alpha-fetoprotein

*VEGF* vascular endothelial growth factor, *VEGFR-2* VEGF receptor-2

*ADAMTS13* a disintegrin-like and metalloproteinase with thrombospondin type 1 motifs 13

VWF, von Willebrand factor; ADAMTS13:AC, ADAMTS13 activity

VWF:Ag, von Willebrand factor antigen; VWF:Ag/ADAMTS13:AC, ratio of VWF:Ag to ADAMTS13:AC

AUC, area under the curve

### Correlation of the tumor volume and stage with the VWF:ag/ADAMTS13:AC ratio and AFP-L3%

We determined the relationship of tumor volume and stage with the VWF:Ag/ADAMTS13:AC ratio and AFP-L3%. Tumor volume and stage significantly correlated with the VWF:Ag/ADAMTS13:AC ratio (*p* = 0.0002 and *p* = 0.046, respectively) but not with the AFP-L3% (Table 4).

**Table 4:** Correlation of the tumor volume and stage with the VWF:Ag/ADAMTS13:AC ratio and AFP-L3%

Variable	Correlation coefficients	*P* value
Tumor volume vs. VWF/ADAMTS13	0.58	0.0002
Tumor stage vs. VWF/ADAMTS13	0.38	0.046
Tumor volume vs. AFP-L3%	0.18	0.91
Tumor stage vs. AFP-L3%	0.28	0.86

HCC, hepatocelullar carcinoma

ADAMTS13, a disintegrin-like and metalloproteinase with thrombospondin type 1 motifs 13

VWF, von Willebrand factor; ADAMTS13:AC, ADAMTS13 activity

VWF:Ag, von Willebrand factor antigen

AFP-L3%, *Lens culinaris* agglutinin-reactive alpha-fetoprotein

VWF:Ag/ADAMTS13:AC, ratio of VWF:Ag to ADAMTS13:AC

### Correlation of serum VEGF levels with the VWF:ag/ADAMTS13:AC ratio and AFP-L3%

**Fig. 5:**
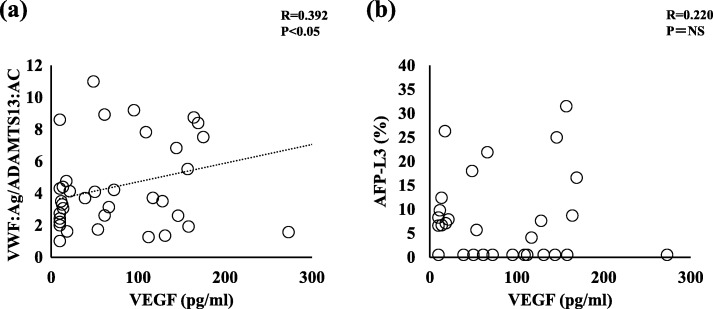
Correlation of the serum VEGF levels with the VWF:Ag/ADAMTS13:AC ratio and AFP-L3%. The serum VEGF level is significantly correlated with the VWF:Ag/ADAMTS13:AC ratio (**a**) but not with the AFP-L3% levels (**b**). VWF, von Willebrand factor; VWF:Ag, VWF antigen; ADAMTS13, a disintegrin-like and metalloproteinase with thrombospondin type 1 motifs 13; ADAMTS13:AC, ADAMTS13 activity; VEGF, vascular endothelial growth factor; AFP-L3%, *Lens culinaris* agglutinin-reactive alpha-fetoprotein

Given that HCC is a highly vascularized tumor, angiogenesis is one of the main contributors to HCC development in which the VEGF signaling pathway plays a pivotal role. Therefore, we investigated whether there was a relationship between the serum VEGF level and the VWF:Ag/ADAMTS13:AC ratio or AFP-L3%. The serum VEGF level was significantly correlated with the VWF:Ag/ADAMTS13:AC ratio (*p* < 0.05) (Fig. 5 a) but not with AFP-L3% (Fig. 5 b).

## Discussion

In recent years, several promising candidate biomarkers for the early detection of HCC have been identified; however, most of these have not been applied for clinical diagnosis due to their high cost and limited practicality in clinical practice [35, 36]. Accordingly, there is a critical unmet medical need to identify novel specific biomarkers for the early detection of HCC. To the best of our knowledge, this was the first report demonstrating that the VWF:Ag/ADAMTS13:AC ratio could serve as a novel biomarker for the early diagnosis of HCC in cirrhotic patients. In the present study, the findings showed that imbalances in the ADAMTS13 enzyme–VWF substrate were associated with HCC development. Moreover, ADAMTS13:AC was reduced in cirrhotic patients with HCC, compared with those without HCC. In contrast, Ikeda et al. revealed that plasma ADAMTS13:AC was higher in patients who developed HCC than in those who did not and that ADAMTS13:AC was an independent risk factor for HCC development [37]. The reason for this different result between the two studies remains unclear. However, one possible explanation is the differences in the progression and the underlying cause of chronic liver disease between the studies. All the patients in our study developed cirrhosis, whereas in the study by Ikeda et al., 43% of the patients had chronic hepatitis [37]. The patients in the present study included those with alcoholic hepatitis, non-alcoholic hepatitis, and autoimmune hepatitis, as well as those with HBV- and HCV-related cirrhosis; whereas only patients with HBV- and HCV-related chronic hepatitis and cirrhosis were included in the study by Ikeda et al. [37]. Furthermore, cirrhotic patients frequently have hypercoagulability, resulting in markedly increased risk for thromboembolism [38, 39]. ADAMTS13 cleaves the VWF in plasma to generate smaller, less thrombogenic fragments. ADAMTS13:AC decreases with increasing severity of liver disease, leading the observed imbalance between the decreased ADAMTS13:AC and the increased VWF:Ag in cirrhotic patients [16, 17]. These results further supported the hypothesis that ADAMTS13 enzyme–VWF substrate imbalances could be related with the hypercoagulability associated with HCC development in cirrhotic patients.

In addition, angiogenesis plays an important role in hepatocarcinogenesis in the early stages. The development of HCC is dependent on the formation of new blood vessels, in which the role of VEGF is critical [40]. The binding of the VWF to integrin avβ3 represses the VEGFR-2 activity and the downstream pro-angiogenic signaling pathways [41, 42]. However, in the current study, VWF:Ag were higher in cirrhotic patients with HCC than in those without HCC. Furthermore, Liu et al. demonstrated that VWF:Ag increased with the progression of chronic hepatitis to HCC [43]. Recently, we found that VWF:Ag increases during the development of HCC [44]. The biological function of VWF depends largely on the size of VWF multimers [45]. Large VWF multimers have been reported to be deficient in malignant diseases [46], indicating a decline in the VWF function. Moreover, the loss of large VWF multimers has been shown to be compensated by an increase in the VWF antigen levels [47]. In addition, ADAM is a fascinating family of secreted transmembrane proteins that function to regulate cell phenotypes through the effects on cell adhesion, migration, proteolysis, and signaling [48]. The levels of ADAM domain-containing protein 28 (ADAM 28) are upregulated in malignant tumors [49], and ADAM 28 derived from cancer cells cleaves and inactivates the pro-apoptotic endogenous agent VWF [50]. These findings suggested that a reduction in the function of VWF results in elevated VWF:Ag in HCC.

ADAMTS13 promotes VEGFR-2 phosphorylation, leading to enhanced VEGF expression and improved angiogenic activity of ECs [51]. In contrast, an in vitro study revealed that when VEGF expression was abundant, ADAMTS13 exerted its anti-angiogenic effects on human ECs [52]. Other studies indicated that VEGF levels progressively increased during the successive stages of hepatocarcinogenesis [40] and that elevated VEGF expression was linked with early-stage HCC [53]. A recent study by Xu et al. revealed the critical role of a balance in the ADAMTS13 enzyme–VWF substrate in regulating blood vessel formation [42]. These findings reiterate the potential role of the VWF:Ag/ADAMTS13:AC ratio in HCC development, suggesting a potential new biomarker that may allow early detection of HCC.

The present study had several limitations, including the absence of clinicopathologic or prognostic data and the small sample size. Patients with liver cirrhosis sometimes develop thrombosis or inflammation, including portal thrombosis, bacterial overgrowth, and translocation. When the VWF:Ag/ADAMTS13:AC ratio is used as a biomarker for the early detection of HCC, thrombosis and inflammation might affect the value of the ratio. Only patients with hypervascular HCCs were analyzed in the present study; therefore, pathologically, early or hypovascular HCC should be examined in the future. Further studies need to be carried out to validate the findings of the present study.

## Conclusion

The VWF:Ag/ADAMTS13:AC ratio probably contributes to HCC development identified small, early-stage, and AFP-negative HCC in cirrhotic patients. Although the use of either VWF:Ag or ADAMTS13:AC alone would be insufficient in aiding the early diagnosis of HCC, the ratio of the two biomarkers can greatly increase the accuracy. The diagnostic accuracy of the VWF:Ag/ADAMTS13:AC ratio was comparable with that of AFP-L3%; nonetheless, the VWF:Ag/ADAMTS13:AC ratio is a remarkably superior diagnostic biomarker of AFP-L3% in terms of universality and objectivity and could serve as a potentially favorable biomarker for the early diagnosis of HCC in cirrhotic patients.

## Data Availability

Raw data were generated at Nara Medical University Hospital. Derived data supporting the findings of this study are available from the corresponding author [T.N] on request.

## Abbreviations

ADAMTS13: a disintegrin-like and metalloproteinase with thrombospondin type 1 motifs 13
ADAMTS13:AC: ADAMTS13 activity
AFP: alpha-fetoprotein
AFP-L3%: *Lens culinaris* agglutinin-reactive fraction of alpha-fetoprotein
AUC: area under the curve
CT: computed tomography
DCP: des-γ-carboxy prothrombin
EC: endothelial cells
ELISA: enzyme-linked immunosorbent assay
HBV: hepatitis B virus
HCC: hepatocellular carcinoma
HCV: hepatitis C virus
HSC: hepatic stellate cells
JSH: Japan Society of Hepatology
LSEC: liver sinusoidal endothelial cells
MRI: magnetic resonance imaging
RFA: radio frequency ablation
SD: standard deviation
UL-VWFM: von Willebrand factor multimers
US: ultrasonography
VEGF: vascular endothelial growth factor
VEGFR-2: VEGF receptor-2
VWF: von Willebrand factor
VWF:Ag: VWF antigen
